# Increased pain in response to mechanical or thermal stimulation in a rat model of incision-induced pain with nicotine dependence and withdrawal

**DOI:** 10.3892/etm.2013.963

**Published:** 2013-02-18

**Authors:** ZONGWANG ZHANG, XIANWEN LIU, SUFEN LU, AILAN YU, ZHIJIAN FU

**Affiliations:** 1 Department of Anesthesiology, Shangdong Province-owned Hospital Affiliated to Shandong University, Jinan 250000;; 2 Department of Anesthesiology, Liaocheng People’s Hospital, Liaocheng 252000;; 3 Jiangsu Province Key Laboratory of Anesthesiology, Xuzhou Medical College, Xuzhou 221001, P.R. China

**Keywords:** nicotine, dependence, withdrawal, incisional pain

## Abstract

The aim of this study was to observe the changes in mechanical withdrawal threshold (MWT) and thermal withdrawal latency (TWL) in a rat model of incisional pain with nicotine dependence and withdrawal. Twelve Wistar rats were randomly divided into a control and a withdrawal group, with 6 rats per group. In the control group, the rats were raised in normal conditions for 7 days without any treatment. A model of plantar incisional pain was established in the right lower extremity and changes in the plantar MWT and TWL of the healthy and operative sides were observed for 7 successive days. In the withdrawal group, the rats were raised in normal conditions and treated with a subcutaneous injection of pure nicotine (3 mg/kg), 3 times each day for 7 days. The model of plantar incisional pain in the right lower extremity was established, and changes in bilateral plantar MWT and TWL were observed for 7 days. The operative side plantar MWT and TWL in the withdrawal group were significantly lower than those in the control group on postoperative days 1–7, respectively (P<0.05). Compared with the healthy side in the control group, the healthy plantar MWT was significantly reduced on postoperative days 1–7 (P<0.05) and TWL was significantly decreased in postoperative days 1–6 (P<0.05) in the withdrawal group. The pain sensitivity to mechanical and thermal stimulation significantly increased in the rat model of incisional pain with nicotine dependence and withdrawal. This is consistent with the clinical increase of postoperative pain observed in patients after quitting smoking.

## Introduction

Worldwide, approximately 1 in 3 adults are smokers (1–3). In developed countries, smoking is the main cause of early mortality in adults. Approximately half of the individuals that have been smoking since adolescence succumb to smoking-related diseases (4). Many simply consider smoking as a bad habit. In fact, it is a type of disease. The World Health Organization (WHO) defines smoking as a chronic addiction disease, and smokers are patients with a chronic disease.

Nicotine is the main component of tobacco. It exhibits pharmacological effects by interacting with nicotinic acetylcholine receptors (nAChRs). Animal experiments and clinical studies have demonstrated that nicotine has analgesic effects (5–7). However, studying the physiological changes in the body following nicotine withdrawal has more practical significance than simply studying its analgesic effect. Nicotine withdrawal causes a number of responses, including convulsion, tremor, bradycardia and depression, as well as neural adaptive changes in glutamate and dopamine receptors and desensitization of nAChRs (8). In a rat model of chronic pain with sciatic nerve injury, the pain sensitivity to mechanical stimulation in nicotine-dependent rats increases, with phosphorylation of cyclic adenosine monophosphate (cAMP)-response element binding protein (CREB) in dorsal horn neurons, activation of microglia and an increase in interleukin (IL)-1β (9,10). In clinical practice, there is a high incidence of back pain in long-term smokers. At 48 h after coronary artery bypass grafting, the dose of opioid drugs for smoking patients is 33% greater than that for non-smoking patients. Female smokers take more opioid drugs for analgesia than non-smokers following gynecological surgery (11). The increased postoperative pain sensitivity for smokers may be related to nicotine dependence caused by long-term smoking and perioperative nicotine withdrawal. However, the mechanism remains unclear. Therefore, the establishment of an animal model of postoperative pain with nicotine dependence and withdrawal to study the mechanism of increased pain sensitivity is extremely important.

Malin *et al* (12–14) established a rat model of nicotine dependence and withdrawal by subcutaneously embedding automatic ALZET^®^ osmotic mini pumps. However, this model has the following problems: i) although a stable plasma nicotine concentration is maintained by the mini pumps, there are no continuous smokers in real life, and ii) the mini pump is expensive. In the present study, a rat model of plantar incisional pain was established based on the previous rat model of nicotine dependence and withdrawal. The changes in pain sensitivity to mechanical and thermal stimulation were observed. This study provides a basis for further investigation of the mechanisms of increased postoperative sensitivity to pain in smokers after quitting smoking.

## Materials and methods

### Animal grouping

Clean healthy male Wistar rats (150–200 g) were provided by the Pharmaceutical Industry Research Institute of Shandong Province, China [SCXK (Lu) 20080002]. This study was carried out in strict accordance with the recommendations in the Guide for the Care and Use of Laboratory Animals of the National Institutes of Health. The animal use protocol has been reviewed and approved by the Institutional Animal Care and Use Committee (IACUC) of Shandong Province-owned Hospital Affiliated to Shandong University. Twelve healthy male Wistar rats were randomly divided into a control and withdrawal group, with 6 rats in each group. In the control group, the rats were raised in normal conditions (room temperature 24±2°C, 50% humidity, circadian rhythm alternation, free food and water) without any treatment. Then, the plantar incisional pain model was created in the right lower extremity and changes in the healthy and operative side plantar mechanical withdrawal threshold (MWT) and thermal withdrawal latency (TWL) were observed for 7 successive days. In the withdrawal group, the rats were fed in normal conditions and treated with a subcutaneous injection of 3 mg/kg pure nicotine (Sigma-Aldrich, St. Louis, MO, USA) 3 times a day (7:00, 15:00 and 23:00) for 7 days. Mecamylamine is a nicotinic antagonist and precipitates nicotine abstinence syndrome in rats. The plantar incisional pain model was then created in the right lower extremity, and the changes in bilateral plantar MWT and TWL were observed for 7 days.

### Establishment of the incisional pain model

The rat model of incisional pain was established according to the method of Brennan *et al* (15). Following anesthetic induction with 2% isoflurane, the anesthesia was maintained using 1.4% isoflurane. Following disinfection with diluted iodine tincture, a 1-cm longitudinal incision (from the plantar proximal end to the toe) was made. The subcutaneous muscle was picked up, followed by longitudinal cutting. The muscle starting site and attachment point were kept intact. After gently pressing for hemostasis, the successive layers of incision were sutured. Wound infection was prevented by the administration of 40,000 units penicillin. The rats were then raised in a quiet and warm environment. Following surgery, there was no dyskinesia in the postoperative foot; however, the rats were unwilling to touch the ground with the foot and licking behavior appeared. This indicated that the incisional pain model was successfully established.

### Determination of MWT

Determination of MWT was performed at 8:00–12:00 using a BME-404 electronic mechanical stimulator (Institute of Biomedical Engineering, Chinese Academy of Medical Sciences, Tianjin, China). The main technical parameters of this equipment were as follows: end face diameter of test needle, 0.6 mm; pressure measurement range, 0.1–50 g; and pressure measurement resolution, 0.05 g. An organic glass box (26×20×14 cm) was placed on the sieve of the metal frame. The rat was placed into the box for 30-min adaptation. The lower extremity plantar surface was touched with the test needle until the escaping behavior appeared. The duration of measurement was 1 min. The pressure value was automatically recorded. The measurement was conducted 5 times for each rat (interval, ≥5 min) and the mean was calculated as MWT for this measurement.

### Determination of TWL

Following MWT measurement, determination of TWL was performed using a BME-410C automatic heat pain stimulator (Institute of Biomedical Engineering). The main technical parameters were as follows: 12 V/10 W halogen lamp; area of stimulating light, <20 mm^2^; timing accuracy, 10 msec; and stimulation temperature, 45–65°C (adjustable). The host equipment was placed on the desktop, with a lamp stand under the table. An organic glass box was placed on the glass plate (thickness, 3 mm). The rat was placed into the box for 30-min adaptation. The lower extremity plantar surface was stimulated with light irradiation until the withdrawal response appeared. The pain latency time was recorded. The measurement was repeated 5 times for each rat (interval, ≥5 min) and the mean was calculated as TWL for this measurement.

### Statistical analysis

Data were expressed as the means ± standard deviation (SD). Statistical analyses were performed using SPSS 13.0 statistical software (SPSS Inc., Chicago, IL, USA). The repeated measures analysis of variance (ANOVA) was performed for comparisons within the groups and a least significant difference (LSD) test was used for comparisons between the two groups. P<0.05 was considered to indicate a statistically significant difference.

## Results

### Variations of MWT in the two groups

**Figure 1:**
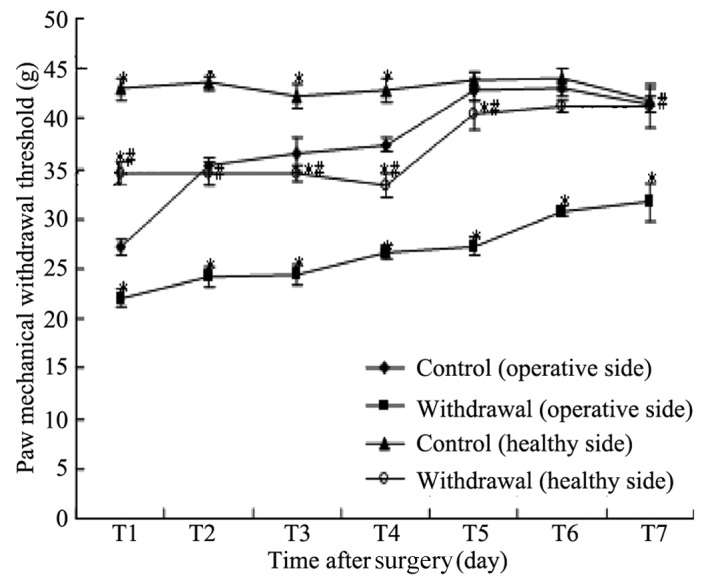
MWT in the two groups on different postoperative days. ^*^P<0.05, compared with the operative side in the control group; ^#^P<0.05, compared with the healthy side in the control group. MWT, mechanical withdrawal threshold.

As shown in Table I and Fig. 1, compared with the operative side plantar MWT in the control group, the operative side plantar MWT in the withdrawal group was significantly lower on postoperative days 1–7, respectively (P<0.05) and the healthy side plantar MWT in the control group was significantly higher on postoperative days 1–4, respectively (P<0.05), with no significant difference in postoperative days 5–7 (P>0.05). Compared with the healthy side plantar MWT in the control group, the healthy side plantar MWT in the withdrawal group was significantly reduced on postoperative days 1–7, respectively (P<0.05).

### Variations of TWL in the two groups

**Figure 2:**
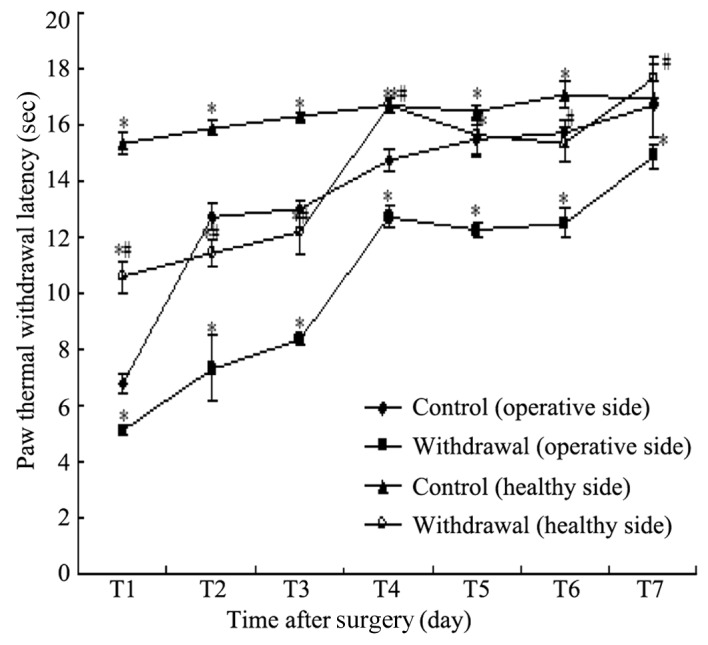
TWL in the two groups on different postoperative days. ^*^P<0.05, compared with the operative side in the control group; ^#^P<0.05, compared with the healthy side in the control group. TWL, thermal withdrawal latency.

TWL in the two groups on different postoperative days are shown in Table II and Fig. 2. Compared with the operative side in the control group, the operative side plantar TWL in the withdrawal group was significantly lower on days 1–7, respectively (P<0.05) and the healthy plantar TWL in the control group was significantly higher on days 1–6 (P<0.05). Compared with the healthy side plantar TWL in the control group, the healthy plantar TWL in the withdrawal group was significantly reduced on days 1, 2, 3, 5 and 6 (P<0.05).

## Discussion

This study identified that the pain sensitivities of the operative and healthy sides to mechanical and thermal stimulation significantly increase in rat models of incisional pain with nicotine dependence and withdrawal. This is consistent with a previous study reporting the increase of postoperative pain sensitivity in patients after quitting smoking (11). However, the exact pathophysiological mechanism of pain modulation by smoking remains unclear.

Over 4,000 different chemical substances have been identified in tobacco. Animal experiments and clinical studies suggest that nicotine is the main component involved in pain modulation (6,16). Exposure to nicotine has significant effects on the central and peripheral nervous systems by combining with nAChRs, which pass through the Na^+^, Ca^2+^ and K^+^ channels. Activation of post-synaptic nAChRs directly acts on excitatory neurons through these cation channels. The activation of presynaptic nAChRs affects the release of other neurotransmitters, including dopamine, glutamate, γ-aminobutyric acid (GABA), 5-hydroxytryptamine (5-HT), histamine and noradrenaline (17). These effects are related to the anti-nociceptive effect of nicotine and the mechanisms involved. Studying the mechanism of pain modulation following nicotine withdrawal has greater clinical significance. Nicotine withdrawal causes numerous emotional responses. In the clinic, various types of chronic pain, including back pain, carpal tunnel syndrome and complex regional pain syndrome, are aggravated in long-term smokers (18–20). After quitting smoking, the postoperative pain is more severe than in non-smoking patients, so more painkillers are required (21,22). These phenomena may be associated with the anti-nociceptive effect of nicotine and the increased pain sensitivity in long-term smoking patients after quitting smoking.

The incisional pain rat model, in which postoperative pain is simulated, was proposed by Brennan *et al* (15). In this model, rats present spontaneous pain, allodynia and thermal hyperalgesia following incisional surgery. At 30 min after surgery, allodynia appears in the foot on the operative side. The allodynia reaches a peak at 2 h and continues for 4–7 days, while the non-operative side is not affected. These pain behaviors and durations of time are similar to clinical intraoperative and postoperative pain. In the current study, the changes in pain behavior in the healthy and operative side planta in the control group are consistent with the above results. The pain sensitivities to mechanical and thermal stimulation in incisional pain rat models with nicotine dependence and withdrawal are significantly higher than in the control. This is consistent with the clinical increase of postoperative pain sensitivity in smokers after quitting smoking.

Long-term exposure to nicotine induces the upregulation and inactivation of nAChRs, resulting in a decrease of inhibitory neurotransmitter release. For patients with acute smoking cessation, the increased pain sensitivity in the first week is related to the elevated utilization of β2^*^-nAChR in the thalamus (23). It was identified that microglia are the responder cells that respond the fastest to injuries, including trauma, ischemia and inflammation. They interact with neurons through chemokines, proinflammatory cytokines, inflammatory mediators, nutritional factors and autoreceptors, participating in the occurrence and development of pain (24). In addition, nAChRs play an important role in the cholinergic anti-inflammatory pathway (25–27). Further studies are required to investigate the correlation of increased pain sensitivity to mechanical and thermal stimulation with activation of microglia in incisional pain rat models with nicotine dependence and withdrawal, as well as the changes in inflammatory mediators in the incision site.

In conclusion, the pain sensitivity to mechanical and thermal stimulation significantly increases in incisional pain rat models with nicotine dependence and withdrawal. This is consistent with the clinical increase of postoperative pain in smokers after quitting smoking and has provided a basis for further investigation into the related pathophysiological mechanisms.

## Figures and Tables

**Table I:** MWT in the two groups on different postoperative days.

Group	Day 1	Day 2	Day 3	Day 4	Day 5	Day 6	Day 7
Control (operative side)	27.25±0.78	35.24±0.81	36.42±1.77	37.38±0.74	42.93±1.09	43.10±0.80	41.46±0.73
Withdrawal (operative side)	22.09±0.89^a^	24.25±1.00^a^	24.48±1.00^a^	26.66±0.74^a^	27.25±0.89^a^	30.83±0.49^a^	31.69±1.91^a^
Control (healthy side)	42.96±1.01^a^	43.58±0.72^a^	42.26±1.27^a^	42.90±1.19^a^	43.92±0.81	43.96±1.08	41.88±1.19
Withdrawal (healthy side)	34.58±1.05^a,b^	34.57±1.19^b^	34.52±0.75^a,b^	33.31±1.24^a,b^	40.41±1.53^a,b^	41.23±0.65^a,b^	41.28±2.16^b^

a P<0.05, compared with the operative side in the control group;

b P<0.05, compared with the healthy side in the control group. Data are presented as means ± standard deviation (SD, g). MWT, mechanical withdrawal threshold.

**Table II:** TWL in the two groups on different postoperative days.

Group	Day 1	Day 2	Day 3	Day 4	Day 5	Day 6	Day 7
Control (operative side)	6.76±0.38	12.74±0.52	13.00±0.32	14.74±0.38	15.49±0.53	15.69±0.51	16.72±0.85
Withdrawal (operative side)	5.12±0.16^a^	7.31±1.17^a^	8.33±0.14^a^	12.69±0.37^a^	12.22±0.27^a^	12.49±0.54^a^	14.85±0.41^a^
Control (healthy side)	15.34±0.40^a^	15.89±0.28^a^	16.33±0.13^a^	16.68±0.25^a^	16.45±0.25^a^	17.09±0.47^a^	16.89±1.34
Withdrawal (healthy side)	10.57±0.56^a,b^	11.43±0.47^a,b^	12.20±0.83^a,b^	16.68±0.24^a^	15.67±0.75^b^	15.32±0.65^b^	17.67±0.74

a P<0.05, compared with the operative side in the control group;

b P<0.05, compared with the healthy side in the control group. Data are presented as means ± standard deviation (SD, sec). TWL, thermal withdrawal latency.

## References

[1] Peto R, Lopez AD, Boreham J, Thun M, Heath C, Doll R (1996). Mortality from smoking worldwide. British Med Bull.

[2] Benowitz NL (2008). Clinical pharmacology of nicotine: implications for understanding, preventing, and treating tobacco addiction. Clin Pharmacol Ther.

[3] Mathers CD, Loncar D (2006). Projections of global mortality and burden of disease from 2002 to 2030. PLoS Med.

[4] Schultz H (1998). Tobacco or health: A global status report. Ann Saudi Med.

[5] Jankowski CJ, Weingarten TN, Martin DP (2011). Randomised trial of intranasal nicotine and postoperative pain, nausea and vomiting in non-smoking women. Eur J Anaesthesiol.

[6] Flood P, Daniel D (2004). Intranasal nicotine for postoperative pain treatment. Anesthesiology.

[7] Anderson KL, Pinkerton KE, Uyeminami D, Simons CT, Carstens MI, Carstens E (2004). Antinociception induced by chronic exposure of rats to cigarette smoke. Neurosci Lett.

[8] Dani JA, Jenson D, Broussard JI, De Biasi M (2011). Neurophysiology of nicotine addiction. J Addict Res Ther.

[9] Brett K, Parker R, Wittenauer S, Hayashida K, Young T, Vincler M (2007). Impact of chronic nicotine on sciatic nerve injury in the rat. J Neuroimmunol.

[10] Josiah DT, Vincler MA (2006). Impact of chronic nicotine on the development and maintenance of neuropathic hypersensitivity in the rat. Psychopharmacology (Berl).

[11] Shi Y, Weingarten TN, Mantilla CB, Hooten WM, Warner DO (2010). Smoking and pain: pathophysiology and clinical implications. Anesthesiology.

[12] Malin DH (2001). Nicotine dependence: studies with a laboratory model. Pharmacol Biochem Behav.

[13] Malin DH, Lake JR, Carter VA (1994). The nicotinic antagonist mecamylamine precipitates nicotine abstinence syndrome in the rat. Psychopharmacology (Berl).

[14] Malin DH, Lake JR, Newlin-Maultsby P (1992). Rodent model of nicotine abstinence syndrome. Pharmacol Biochem Behav.

[15] Brennan TJ, Vandermeulen EP, Gebhart GF (1996). Characterization of a rat model of incisional pain. Pain.

[16] Simons CT, Cuellar JM, Moore JA (2005). Nicotinic receptor involvement in antinociception induced by exposure to cigarette smoke. Neurosci Lett.

[17] LeSage MG, Keyler DE, Shoeman D, Raphael D, Collins G, Pentel PR (2002). Continuous nicotine infusion reduces nicotine self-administration in rats with 23-h/day access to nicotine. Pharmacol Biochem Behav.

[18] (2002). Nicotine and caffeine intake in complex regional pain syndrome. J Back Musculoskelet Rehabil.

[19] Nathan PA, Meadows KD, Istvan JA (2002). Predictors of carpal tunnel syndrome: an 11-year study of industrial workers. J Hand Surg Am.

[20] Nyiendo J, Haas M, Goldberg B, Sexton G (2001). Pain, disability, and satisfaction outcomes and predictors of outcomes: a practice-based study of chronic low back pain patients attending primary care and chiropractic physicians. J Manipulative Physiol Ther.

[21] Yang Z, Arheart KL, Morris R (2012). CYP2D6 poor metabolizer genotype and smoking predict severe postoperative pain in female patients on arrival to the recovery room. Pain Med.

[22] Woodside JR (2000). Female smokers have increased postoperative narcotic requirements. J Addict Dis.

[23] Cosgrove KP, Esterlis I, McKee S (2010). Beta2^*^ nicotinic acetylcholine receptors modulate pain sensitivity in acutely abstinent tobacco smokers. Nicotine Tob Res.

[24] Kreutzberg GW (1996). Microglia: a sensor for pathological events in the CNS. Trends Neurosci.

[25] De Simone R, Ajmone-Cat MA, Carnevale D, Minghetti L (2005). Activation of alpha7 nicotinic acetylcholine receptor by nicotine selectively up-regulates cyclooxygenase-2 and prostaglandin E2 in rat microglial cultures. J Neuroinflammation.

[26] Libert C (2003). Inflammation: A nervous connection. Nature.

[27] Wang H, Yu M, Ochani M (2003). Nicotinic acetylcholine receptor alpha7 subunit is an essential regulator of inflammation. Nature.

